# Supplementary material for: Prevalence of violence in a clinical sample of adolescent patients visiting a child and adolescent psychiatric outpatient clinic in Nepal
Source: PLoS One. 2025 Oct 31;20(10):e0335396. doi: 10.1371/journal.pone.0335396 (PMC12578155; doi:10.1371/journal.pone.0335396)
Supplement: Attachment — Submitted filename: PONE-D-25-20724.docx [file pone.0335396.s003.docx]

**Introduction**

The introduction is well-founded, supported by global data that effectively justifies the need for this study. Excellent work.

**Methods and Materials**

- Under **“Study Site and Population”**, include the following statement regarding ethical approval:

*“This study was approved by the Kanti Children's Hospital Ethical Review Committee (Reference No: 621) and the Nepal Health Research Council (Reference No: 1227).”*

- In the **“Procedure”** section, remove the following sentence from the end of the paragraph:
  *“All participating adolescents received a small gift as a token of their help.”*

**Results**

- In **Table 1 – Distribution of Selected Socio-Demographic Variables**, organize the data in descending order based on the absolute number (N). See the example below for formatting guidance:

| **Variables** | **N** | **%** |
| --- | --- | --- |
| Middle Hills (including Kathmandu) | 654 | 80.7 |
| Southern Plains/Terai | 134 | 16.5 |
| Himalayan | 22 | 2.7 |

**Discussion**

Enhance the discussion related to the **prevalence of violence in a clinical sample of adolescent patients attending a child and adolescent psychiatric outpatient clinic**. The fact that **88% of adolescents reported experiencing some form of violence** is highly significant. This finding suggests that a substantial proportion of these adolescents may be seeking psychiatric care due to the psychological consequences of violence exposure. Although this relationship was not directly examined in the current study, it is important to highlight this point in the discussion section. Doing so would raise awareness among readers about the potential psychiatric symptoms and mental health outcomes frequently associated with experiences of violence in adolescence.

**Conclusions**

Clearly emphasize the **prevalence of violence in a clinical sample of adolescent patients attending a child and adolescent psychiatric outpatient clinic in Nepal**, underlining that **88% of participants had experienced some form of violence**. This finding reinforces the urgent need for integrated mental health and psychosocial interventions for this vulnerable population.
